# Neonatal hereditary spherocytosis caused by a de novo frameshift mutation of the *SPTB* gene characterized by hydrops fetalis: A case report

**DOI:** 10.1097/MD.0000000000024804

**Published:** 2021-03-26

**Authors:** Yimin Zhang, Shuming Shao, Jie Liu, Chaomei Zeng, Ye Han, Xiaorui Zhang

**Affiliations:** Department of Pediatrics, Peking University People's Hospital, Beijing, China.

**Keywords:** case report, hereditary spherocytosis, hydrops fetalis, neonate, *SPTB* gene

## Abstract

**Rationale:**

The etiology of non-immune hydrops fetalis is complex, and its prognosis is poor. One of its main causes is anemia. There are few reports on hydrops fetalis due to anemia caused by hereditary spherocytosis (HS), especially regarding its occurrence in the neonatal period. Thus, we report on a case of neonatal HS caused by a new *SPTB* gene mutation that was characterized by hydrops fetalis.

**Patient concerns:**

A neonate with intrauterine hydrops fetalis showed severe hyperbilirubinemia and anemia, reticulocytosis, and hepatosplenomegaly. Laboratory examination findings were normal.

**Diagnoses:**

Gene sequencing of the patient and his parents showed a de novo frameshift mutation in the patient's *SPTB* gene. Ultimately, the patient was diagnosed with HS.

**Interventions:**

Exchange and red blood cell transfusions were performed in the neonatal period.

**Outcomes:**

The child was discharged from the hospital 14 days postnatal because his hemoglobin and bilirubin levels were stable. Red blood cell transfusion was performed once in infancy; however, no further red blood cell transfusions were required within 2 years of age.

**Lessons:**

Hydrops fetalis can be a manifestation of HS. Genetic detection can help confirm the diagnosis of suspected neonatal HS undocumented by other laboratory examinations.

## 1 Introduction

Hydrops fetalis is characterized by excessive accumulation of fetal extracellular fluid, which manifests as 2 or more abnormal fetal body cavity effusions (including the thoracic, abdominal, and pericardial cavities) and can lead to fetal and neonatal deaths. Hydrops fetalis is divided into immune and non-immune hydrops fetalis; non-immune hydrops fetalis accounts for more than 90% of all cases, and the incidence rate is approximately 1 to 3 per 1000 births.^[1]^ The etiology of non-immune hydrops fetalis is complex and its prognosis is poor; therefore, personalized diagnostic methods and treatment programs according to the different causes should be used in clinical practice. One of the main causes of hydrops fetalis is anemia, accounting for approximately 10% to 27% of all cases. Currently, the main causes of the anemias that lead to hydrops fetalis are severe alpha-thalassemia, parvovirus infection, and maternal blood transfusion, and so on^[2]^; while hydrops fetalis due to anemia caused by hereditary spherocytosis (HS), especially that occurring in the neonatal period, is rarely reported.^[3–8]^ Among these reports, Hannah et al^[3]^ and Chonat et al^[4]^ reported 2 cases of hydrops fetalis caused by HS due to an *SPTA1* gene mutation, and Gallagher et al^[6,7]^ reported 1 case of hydrops fetalis caused by HS due to a β-hemoglobin abnormality. Herein, we report on a case of neonatal HS caused by a new *SPTB* gene mutation and characterized by hydrops fetalis.

This case report was prepared following the CARE guidelines. Written informed consent was obtained from the parents of the baby patient for his anonymized clinical and genetic data to be analyzed and published for research purposes.

## 2 Case report

A male patient was born by caesarean delivery due to hydrops fetalis at a gestational age of 33 weeks and 6 days and weighed 2640 g at birth. He was the first child of his non-consanguineous, 37-year-old, gravida 2 para 1 mother, and 35-year-old father. According to the father, their first pregnancy was aborted for personal reasons. The mother's blood type was type A Rhesus positive; she had regular prenatal examinations during pregnancy and non-invasive prenatal testing classified her as low risk. The ultrasound at 23 weeks of gestation showed a small amount of pleural effusion on the right side of the fetus, and dynamic ultrasound monitoring conducted 1 to 2 times per week showed that the amount of pleural effusion had gradually increased. Hydrops fetalis was diagnosed at 29 weeks of gestation, manifesting as fetal pleural and peritoneal effusions. His parents were born in Beijing and denied a family genetic history.

After delivery, the patient was cyanotic, moaning with xiphoid retractions, and had dyspnea that was quickly relieved by closed thoracic drainage and respiratory support. However, he developed anemia and significant jaundice within 12 hours postnatally.

Physical examination showed no physical malformations or cardiac murmurs; however, he had hepatosplenomegaly. The liver was palpable 3.5 cm below the costal margin and 3 cm below the xiphoid process, and the spleen was palpable 2 cm below the costal margin. Two hours postnatally, the laboratory examination showed a hemoglobin level and reticulocyte rate of 145 g/L and 11.5%, respectively. Twelve hours postnatally, the hemoglobin level decreased to 120 g/L and the total bilirubin level was 138.5 μmol/L. Further laboratory examination revealed that the child's blood type was type O Rhesus positive, and the Coombs test was negative. His routine blood, urine, and stool test results; C-reactive protein; and blood culture were normal. He was negative for hepatitis B surface antigen; hepatitis C antibody; Treponema pallidum particle agglutination; and toxoplasmosis, rubella cytomegalovirus, herpes simplex, human immunodeficiency virus, parvovirus B19, and Epstein-Barr virus antibodies. Despite intense phototherapy and an albumin infusion, the jaundice worsened, and 60 hours postnatally, the total bilirubin level was 430.9 μmol/L. Following blood exchange therapy, the bilirubin level decreased to normal.

**Figure 1:**
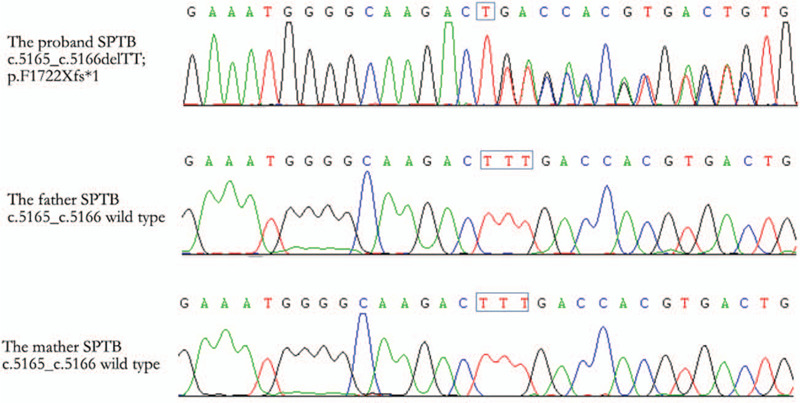
Sequencing diagram verification of SPTB mutations.

As there was a high suspicion of non-immune hemolytic anemia, peripheral blood smear, osmotic fragility testing, and glucose-6-phosphate dehydrogenase and hemoglobin electrophoreses were conducted at 12 hours postnatally; however, all the results were negative, which was not consistent with the clinical manifestations. The eosin-5-Maleimide binding test was not conducted because the reagents were not available and, after obtaining their consent, whole exon gene sequencing of the entire family was performed to assist in the diagnosis. The results of the sequencing showed that there was a frameshift mutation in the *SPTB* gene of the child, which was c.5165_c.5166delTT (Fig. 1). This was diagnosed as HS. Since the *SPTB* gene mutation was not detected in the parents, the mutation was a heterozygous de novo mutation in the proband, with deletion of 2 T bases at exon 5165 and 5166 of the gene 24, resulting in the termination of the encoded protein from phenylalanine at position 1722.

On the 14th day postnatally, his hemoglobin and total bilirubin levels (95 g/L and 137.3 μmol/L, respectively) were stable and he was discharged. At the age of 42 days, his hemoglobin level, reticulocyte ratio, and total bilirubin level were 64 g/L, 0.097, and 66.5 μmol/L, respectively; thus, he was readmitted to the hospital for red blood cell infusion. His hemoglobin level was 85 to 105 g/L at the ages of 2 to 24 months, and he did not undergo transfusion again.

## 3 Discussion

Among the neonates listed in the United States of America Kernicterus Registry, HS was the third most common underlying hemolytic condition after glucose-6-phosphate dehydrogenase deficiency and ABO hemolytic disease. Herein, we described a case of neonatal HS caused by a newly discovered *SPTB* gene mutation characterized by hydrops fetalis and severe postnatal hyperbilirubinemia. According to the American Society of Medical Genetics and Genomics guidelines, the nature of the gene variation in this case relates to very strong pathogenicity evidence (PVS1) + strong pathogenicity evidence (PS2) + moderate pathogenicity evidence (PM2), which is a combination of the “pathogenic” variation; thus, the nature of the variation is determined as pathogenic. Based on a search of the Online Mendelian Inheritance in Man and Clinvar databases and Human Gene Mutation Database, we found no disease-related reports. Moreover, no reports were found in the normal human databases (DYDF, Single Nucleotide Polymorphism Database, 1000 genomes, Exome Aggregation Consortium). Because his parents had normal phenotypes and no *SPTB* gene mutations, the mutation was considered a new heterozygous mutation of the proband, in accordance with the incidence law of autosomal dominant genetic diseases. The risk of HS in future offspring is low.

Previous studies have shown that jaundice is the main manifestation of neonatal onset HS.^[9,10]^ The index case had extremely severe hyperbilirubinemia after birth, which was corrected with intensive phototherapy and an exchange transfusion. Therefore, the bilirubin level in newborns in whom HS is suspected should be monitored closely and treated promptly. Anemia and splenomegaly are not obvious in neonates with HS, which is consistent with the findings of this case. The hemoglobin levels of children with HS can be within normal ranges at birth and can remain normal 1 week postnatally. However, with the occurrence of hemolysis and limited red blood cell production in newborns, they may gradually develop anemia or may even develop severe anemia. Most children rely on red blood cell transfusions within the first year of life but do not require them thereafter. When considering the diagnosis of HS in newborns with non-immune hemolytic anemia, the search for spherocytes on peripheral blood smears, and the eosin-5-Maleimide binding and erythrocyte osmotic fragility tests aid in the diagnosis.

This patient had no related family history, no spherocytes were found on the peripheral blood smears, and the osmotic fragility test result was negative on admission, which did not support the HS diagnosis. However, anemia and severe hyperbilirubinemia developed postnatally, hepatosplenomegaly was detected on examination, the proportion of reticulocytes was significantly increased, and the hemoglobin level decreased gradually; these findings were suggestive of extramedullary hematopoiesis. Finally, an *SPTB* gene frameshift mutation was found on gene sequencing. It may have been related to the absence of obvious spherocytes, relatively low osmotic fragility test sensitivity and specificity, and specific HS expression that is associated with a new gene variation in one-third of newborns with HS. Therefore, in clinical practice, when severe non-immune hemolytic anemia with a negative family history occurs, even if there are no positive findings with the laboratory examination, the possibility of neonatal non-immune hemolytic diseases such as HS should not be ignored, and gene sequencing should be considered to assist in diagnosis, if necessary.

## Acknowledgments

The authors would like to extend our thanks to all participants in this study.

## Author contributions

**Conceptualization:** Yimin Zhang, Xiaorui Zhang.

**Data curation:** Yimin Zhang, Shuming Shao.

**Formal analysis:** Shuming Shao, Ye Han.

**Investigation:** Yimin Zhang, Shuming Shao.

**Methodology:** Yimin Zhang, Ye Han.

**Resources:** Yimin Zhang, Xiaorui Zhang.

**Supervision:** Jie Liu, Chaomei Zeng.

**Writing – original draft:** Yimin Zhang.

**Writing – review & editing:** Jie Liu, Chaomei Zeng, Xiaorui Zhang.
